# Organic Salts and Merrifield Resin Supported [PM_12_O_40_]^3−^ (M = Mo or W) as Catalysts for Adipic Acid Synthesis

**DOI:** 10.3390/molecules24040783

**Published:** 2019-02-21

**Authors:** Jana Pisk, Dominique Agustin, Rinaldo Poli

**Affiliations:** 1 Centre National de la Recherche Scientifique (CNRS), Laboratoire de Chimie de Coordination (LCC), Université de Toulouse, UPS, INPT, 205, route de Narbonne, 31077 Toulouse, France; rinaldo.poli@lcc-toulouse.fr; 2 Université de Toulouse, IUT P. Sabatier, Département de Chimie, Av. G. Pompidou, BP 20258, 81104 Castres CEDEX, France; 3 Institut Universitaire de France, 103, bd Saint-Michel, 75005 Paris, France

**Keywords:** polyoxometalates, Merrifield resin, cyclohexene, adipic acid, organic solvent-free process

## Abstract

Adipic acid (AA) was obtained by catalyzed oxidation of cyclohexene, epoxycyclohexane, or cyclohexanediol under organic solvent-free conditions using aqueous hydrogen peroxide (30%) as an oxidizing agent and molybdenum- or tungsten-based Keggin polyoxometalates (POMs) surrounded by organic cations or ionically supported on functionalized Merrifield resins. Operating under these environmentally friendly, greener conditions and with low catalyst loading (0.025% for the molecular salts and 0.001–0.007% for the supported POMs), AA could be produced in interesting yields.

## 1. Introduction

**Scheme 1:**
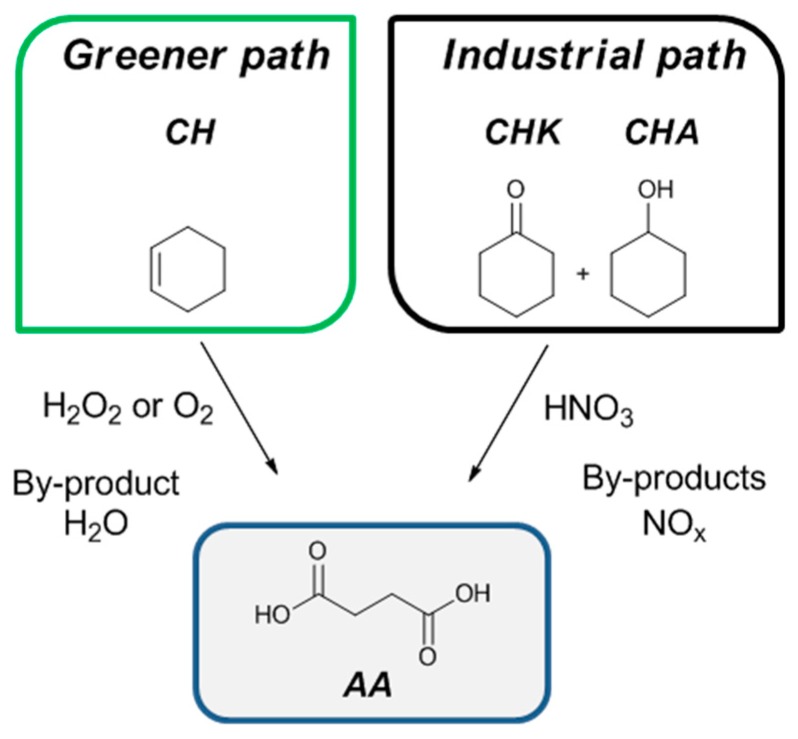
Proposed routes for the synthesis of adipic acid (AA).

Adipic acid (AA) is one of the reagents used for the production of nylon-6,6, a polymer that exhibits high mechanical strength, rigidity, and good thermal and chemical stability [1]. Most industrial processes for the AA production (2.3 Mtons/year) involve nitric acid oxidation of cyclohexanol (CHA) and/or cyclohexanone (CHK) [2,3]. This inexpensive acid, used in several industrial processes [4], however, leads to pollution, producing unwanted waste and various nitrogen oxides (NO_x_), including ca. 400 ktons of the greenhouse gas N_2_O per year [5,6]. Although this emission can be significantly reduced using a thermal decomposition process [7,8], current academic and industrial process developments focus on cleaner technologies [2,3,9,10,11]. One possible alternative route for AA production is the selective oxidation of cyclohexene (CH) with hydrogen peroxide (H_2_O_2_) [12,13] or molecular oxygen (O_2_) [14] as preferred oxidants (Scheme 1). Even though recent reports describe a very fast AA production under high pressure and high temperatures conditions [15,16,17,18,19], using membrane reactors [20] or microwave conditions [21], our research goal is to apply user-friendly and safe protocols (lower temperatures avoiding the H_2_O_2_ decomposition, atmospheric pressure, one step reaction, recoverable catalyst), and to compare them with the classical protocols used in industrial processes.

Heteropolyoxometalates—inorganic compounds with general chemical formula [X*_n_*M*_p_*O*_q_*]*^z^*^−^, M being commonly Mo or W, and X being usually a main group element (Si, P, B)—were described as suitable for catalyst design at the atomic and molecular levels [22]. Among all structures, the one corresponding to the discrete assembly of 12 M around one X, with a general formula [XMo_12_O_40_]^n−^ (called Keggin), is very stable. Different isomers of Keggin polyoxometalates (POMs) can exist, according to the arrangement of the metal oxides around the heteroatom. Keggin-type POMs are efficient catalysts for liquid phase oxidation [23], due to their stability towards oxygen donors and their ability to incorporate different transition metals [24]. Immobilization of POMs through electrostatic interactions in insoluble polymer resins can lead to easier separation and recovery of the molecular catalysts [25,26]. Other interesting supports are SiO_2_ [27], mesoporous silica (SBA) [28,29] or certain macroporous resins [30]. Among the resins, the low-cost and relatively robust Merrifield resins (MR), originally developed for the solid-state synthesis of peptides [31], have the ability to undergo facile functionalization [25]. Even though many transition metal catalysts have been supported on MR for catalytic purposes [25,32,33], there is no report, to the best of our knowledge, on the application of such ionically supported POM-based catalysts for AA production.

The AA production from a mixture of CHA and CHK (so-called KA “ketone-alcohol” oil [34]) has previously been described using various homogeneous catalysts such as H_2_WO_4_ [35] or Keggin-based POMs, i.e., M_x_PMo_12_O_40_ (M = Ni, Co) [36], H_3−2x_Co_x_PMo_12_O_40_ [37], or Y_3−2x_Ni_x_PMo_12_O_40_ (Y = H or NH_4_, x = 0.25–1.5) [38]. With (C_4_H_9_N)_3_[PMo_12_O_40_], AA (18% yield) and CHK were produced from CHA, while (C_4_H_9_N)_3_[PW_12_O_40_] produced CHK only [39]. The replacement of the substrates (CHA, CHK) used in industrial processes by other starting substrates (CH, cyclohexane oxide CHO, 1,2-cyclohexanediol CHD), is guided by several economical parameters (substrate price, target yield, availability, time cost analysis, etc.). An attractive starting material, CH, was successfully converted to AA by aqueous H_2_O_2_ using Na_2_WO_4_ as a catalyst and the [CH_3_(*n*-C_8_H_17_)_3_N]HSO_4_ phase transfer agent [40]. The oxidation of *trans*-CHD is another suitable pathway for AA production [3,10,41,42] providing the highest AA yields but CHD is the most expensive reagent among the listed substrates. On the other hand, CH is largely available by benzene hydrogenation [43,44,45] or by cyclohexane dehydrogenation [46,47,48] and, therefore, less expensive and more promising. Since CHD can be obtained by the oxidation of CH, a more direct and selective AA “one-pot” production from CH is of great interest.

Different heterogeneous catalysts based on calcined POMs [49], mesoporous solids [50], Ti-MMM-2 [51], Ce-SBA-15 [51], TAPO-5 [52], and Ti-AlSBA-15 [53,54] were also tested for direct CH oxidation, the AA yield being improved by the stepwise addition of H_2_O_2_ to the reaction mixture. The catalytic mechanism for the CH oxidation into AA through CHD was clarified with TAPO-5 as a catalyst [52], showing that *trans*-CHD was formed from cyclohexene oxide (CHO) while the *cis*-CHD formation followed a radical mechanism. The CH oxidation to AA was also tested in CH_3_CN using TBHP (*tert*-butylhydroperoxide) in decane as oxidant and Ti-AlSBA-15 as a catalyst [54]. Some literature data and reaction conditions with CH, CHO, CHD, CK, and CHA substrates are listed in Table 1.

In the present contribution, we report the preparation and characterization of new molecular and resin-supported Mo- and W-based Keggin-type catalysts and their catalytic performance for the production of AA starting from CH. The main objective of the presented research was to evaluate a greener process, avoiding the use of organic solvent, using a new family of grafted catalysts and minimizing the catalyst loading. Different starting substrates for AA production were tested, in order to gain a better insight into the oxidation mechanism, especially by comparison between molecular and grafted catalysts.

## 2. Results and Discussion

### 2.1. Synthesis and Characterization of the Prepared Catalysts

#### 2.1.1. Synthesis

**Scheme 2:**
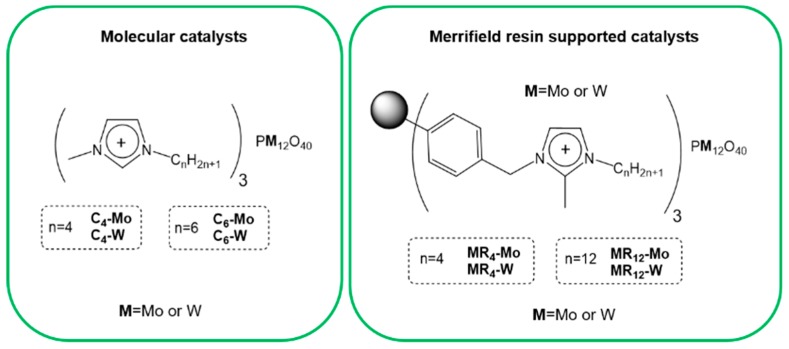
Catalysts used.

Four molecular catalysts, (Scheme 2, left), were straightforwardly prepared by the reaction of the corresponding ionic liquid (1-hexyl-3-methylimidazolium bromide or 1-butyl-3-methylimidazolium bromide) and phosphomolybdic or phosphotungstic acid. The four isolated complexes are composed by a [PM_12_O_40_]^3−^ (M = Mo or W) surrounded by three alkylmethylimidazolium cations.

**Scheme 3:**
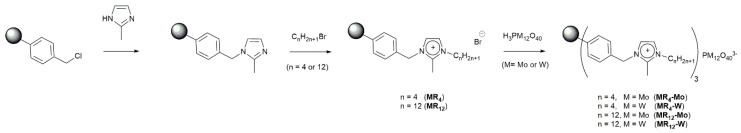
Synthetic procedure for the preparation of the heterogeneous catalysts.

The synthesis of the polymer-supported catalysts (Scheme 2, right) was achieved as shown in Scheme 3. Merrifield resins were used as polymeric supports. The resins were modified to obtain two different types of imidazolium-functionalized supports (**MR_4_** or **MR_12_**) in two steps (see Scheme 3). The first step consisted of the addition of 2-methylimidazole in the presence of a base to the Merrifield resin, leading to the **MR_Im_** [59]. The imidazole-functionalized intermediate **MR_Im_** was further treated in the second step with the appropriate alkyl bromide to obtain the desired imidazolium moieties (**MR_4_** and **MR_12_**). The different alkyl chain lengths were expected to increase the lipophilicity of the catalytic object, enhancing the catalyst compatibility with the apolar substrate relatively to the water present in the reaction media since carried by the oxidant (H_2_O_2_ in water). In the last step, the POMs ([PMo_12_O_40_]^3−^ or [PW_12_O_40_]^3−^) were then immobilized by simple ion exchange adding the corresponding heteropolyacids (H_3_[PMo_12_O_40_] or H_3_[PW_12_O_40_]). All the synthesized catalysts were characterized by FTIR and elemental analysis.

#### 2.1.2. Characterization

• Infrared (IR) Spectroscopy

**Figure 1:**
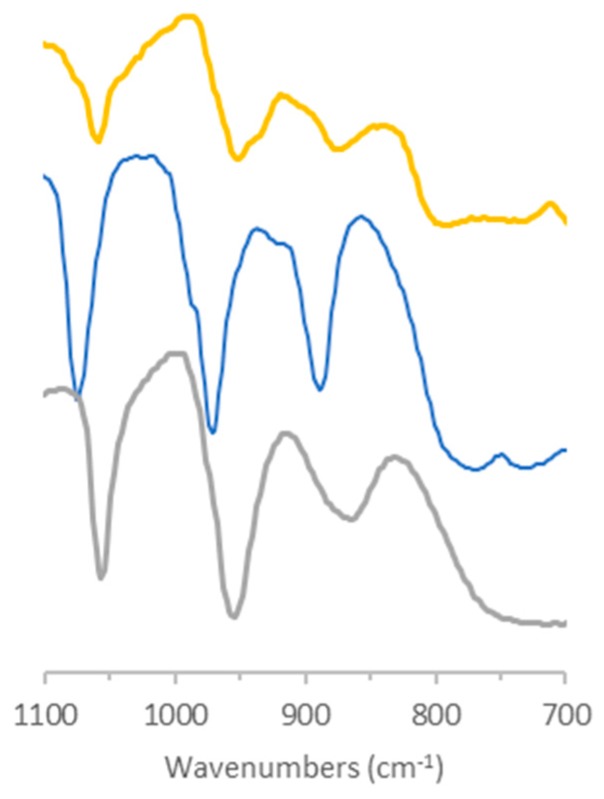
Infrared (IR) spectra of the zone of POM vibrations for the commercial H_3_PMo_12_O_40_ precursor (grey), **C_6_-Mo** molecular catalyst (blue) and **MR_12_-Mo** grafted catalyst (orange).

The IR spectra of the molecular catalysts **C_n_-M** (n-4,6; M-Mo, W) were assigned according to the literature data [60,61,62,63]. The main characteristic features of the Keggin structure were observed at 1078 cm^−1^ (ν_as_ P−O_a_), 970 cm^−1^ (ν_as_ M−O_d_), 885 cm^−1^ (ν_a_ M−O_d_−M), and 771–777 cm^−1^ (ν_as_ M−O_c_−M) for both M (W and Mo). For the catalysts grafted on Merrifield resins **MR_n_-M** (*n* = 4, 12; M-Mo, W), the IR analysis showed the characteristics bands of the 2-methylimidazolium cation, 1420–1490 cm^−1^. The final POM-containing resins exhibit characteristic IR bands at 1060, 955, 880, and 795–802 cm^−1^ for **MR_4_-Mo** and **MR_12_-Mo**, and at 1080, 975, 890, and 795–810 cm^−1^ for **MR_4_-W** and **MR_12_-W**, corresponding to the Keggin anions (Figure 1 shows the specific area corresponding to the POM backbone for **C_6_-Mo**, **MR_12_-Mo** and the corresponding starting H_3_[PMo_12_O_40_], proving that the POM structure remains unchanged when grafted).

• ^31^P Nuclear Magnetic Resonance (NMR) Spectroscopy

**Figure 2:**
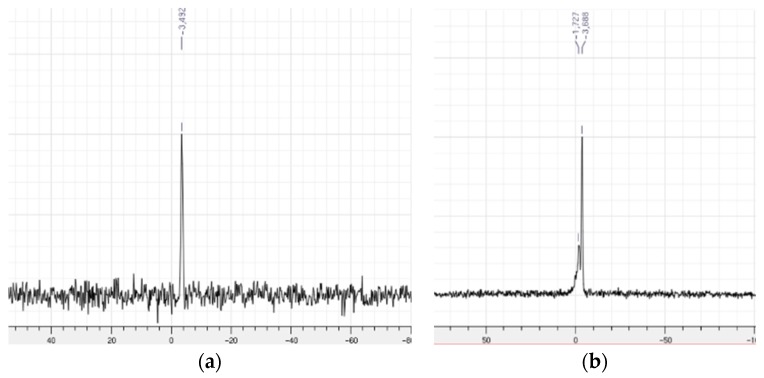
Solid-state ^31^P NMR of **MR_4_-Mo** (**a**) and **MR_12_-Mo** (**b**) supported catalysts.

**Figure 3:**
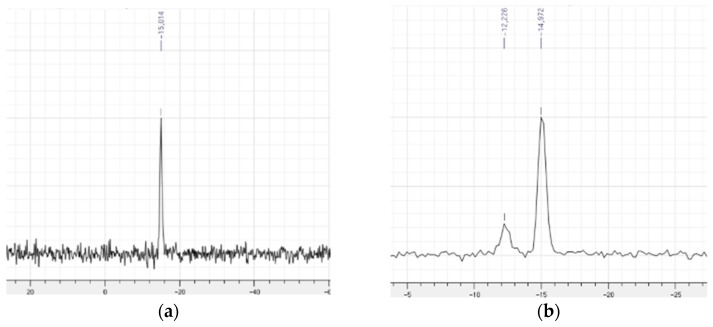
Solid-state ^31^P NMR of **MR_4_-W** (**a**) and **MR_12_-W** (**b**) supported catalysts.

The ^31^P NMR spectra of **C_4_-Mo** and **C_6_-Mo** salts in the solution exhibit a characteristic peak at −4.15 ppm, while those of **C_4_-W** and **C_6_-W** salts show a peak at −15.5 ppm, in accordance with the literature data [64,65]. In the solid-state ^31^P NMR spectrum of the Mo-grafted catalysts (Figure 2), the resonance is shifted to −3.5 ppm in case of **MR_4_-Mo**, while two resonances at −1.9 and −3.5 ppm are observed for **MR_12_-Mo**. Likewise, in the case of the W catalysts (Figure 3), one resonance is observed for **MR_4_-W** (at −15.1 ppm) and two for **MR_12_-W** (at −12.4 and −15.2 ppm). The similar behavior of both POMs seems to depend on the length of the imidazolium substituent and not on the quality of the Keggin POM precursor (the ^31^P NMR in D_2_O of the commercial Mo and W precursors displayed only one signal at −3.9 and −15.3 ppm, respectively). The observed behavior indicates the presence of different cation-dependent interactions at the resin surface, the two simultaneously observed species possibly corresponding to species located in two different sites (i.e., closer to or farther from the imidazolium moiety) [66,67].

• Elemental and Thermogravimetric Analyses

The EA data of the grafted resins show that 32% of Cl from the –CH_2_Cl pending functions has been replaced by the 2-methylimidazolium function.

**Figure 4:**
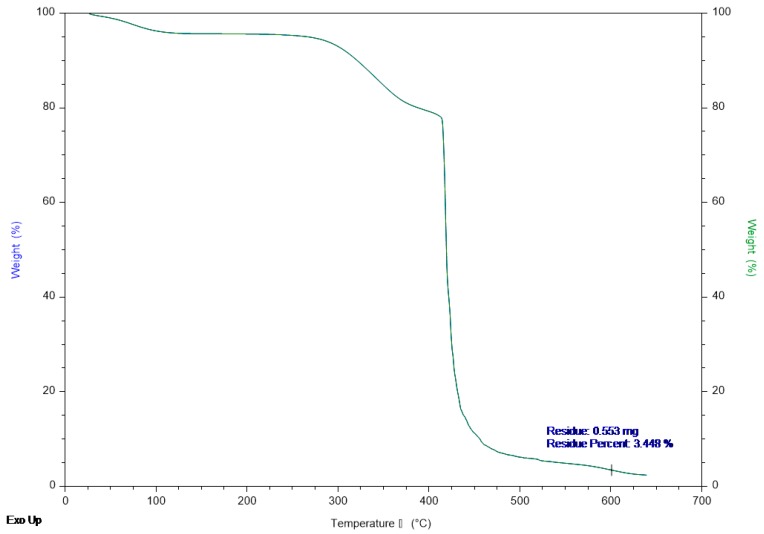
Thermogravimetric curve of **MR_4_-W**.

The metal loadings were determined from TG analyses. The residue after thermal treatment was identified as P_2_O_5_ and MO_3_ (M = Mo or W), see Table 2. Figure 4 shows an illustrative example of the TG curve for **MR_4_-W**.

### 2.2. Catalysis

**Scheme 4:**
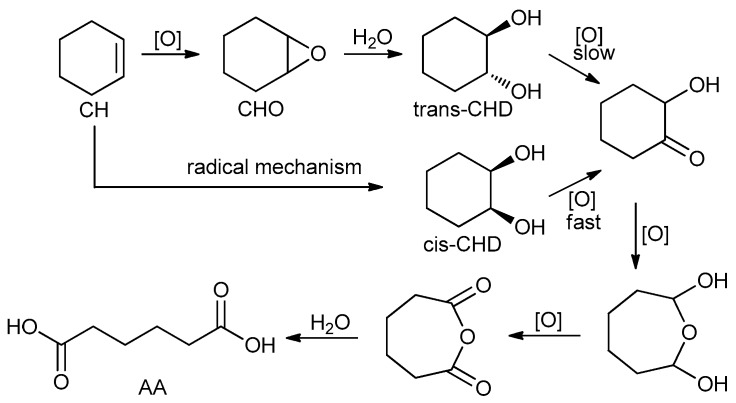
Steps for the oxidation of CH to AA in the presence of TAPO-5 catalyst. Adapted from [52].

The transformation of CH to AA is reported to involve olefin epoxidation, alcohol oxidations, and Baeyer-Villiger oxidation (Scheme 4) [52].

For this reason, we studied the AA formation from different starting compounds (CH, CHO, CHD), using molecular and grafted catalysts. CHO and CHD were used as substrates since they are intermediates in the formation of AA from CH. The formation of the CHD intermediate has also been monitored when starting from CH or CHO (see results in Table 3).

• Swelling Tests

In order to verify whether all the MR-supported POMs are accessible to the substrates under organic solvent-free conditions, swelling tests were performed on non-treated MR. Heating the MR in the presence of CH or CHO led to a mass increase by ca. 2% in the case of CH and 8% in the case of CHO, indicating substrate incorporation into the polymer pores and, therefore, catalyst accessibility without organic solvent. This feature is of great importance since it justifies the use of MR as a support in the catalytically solvent-free processes.

• Classical Conditions

Initial tests were performed with the molecular catalysts **C_n_-M** (n = 4 or 6, M = Mo or W; 0.025 mol% vs. substrate), i.e., a 40 times lower catalyst loading than that reported for Na_2_WO_4_ [40]. All the catalysts were insoluble in the reaction media, but became partially soluble as the reactions evolved, better solubilization was observed for the W-based ones.

• CH as the Substrate

After 60 min, the CH conversion was between 7 and 10% (analyzed by GC) with a low selectivity towards CHO (3%). After 6.5 h, all the molecular catalysts provided a 5–8% selectivity towards CHO with a CH conversion between 12 and 18%. No CHD was observed in the organic layer. The slow conversion is certainly due to the low catalyst loading and to the mild reaction conditions compared to those described in the reference study [35]. After 24 h, however, reasonable conversions were achieved and CHO was no longer detected by GC whereas CHD was present (see Table 1). AA was isolated as a solid in relatively good yields. As stated in several mechanistic studies [52], the species unidentified by GC might correspond to the product of Baeyer Villiger oxidation (see Scheme 4). The observation that CHO is formed and then consumed indicates that this is involved in the AA formation mechanism, via the conversion to CHD.

The homogeneous **C_n_-W** catalysts were more active than the corresponding **C_n_-Mo**, with AA isolated yields ca. 10% higher on average (see Table 1). Control experiments without catalyst or in the presence of the commercial MR yielded only a very low amount of cis and trans-CHD (<7%) after 24 h. The shorter carbon chain on the imidazolium cation afforded better results. The same trend was previously observed for the same reaction with (Hgly)_3_[PM_12_O_40_] (M = Mo, W; gly = glycine) [68] or for the cyclooctene epoxidation with alkylpyridinium salts of [PMo_12_O_40_]^3−^ [61]. The stirred biphasic reaction medium appeared as an emulsion, possibly stabilized by the solid POM salt (Pickering-type) [69,70,71,72].

For the grafted version of the catalysts, the W-based were again more active, despite the lower W content in the case of **MR_12_-W** than for **MR_12_-Mo** (see Table 1). In contrast to the molecular catalysts, the longer chain on the imidazolium cation led to greater activity. This could be linked to a better “solubilization” of the substrate near the catalyst active site.

• CHO as the Substrate

Complete substrate conversion was observed in this case, with the molecular or the grafted catalysts, producing AA after one day in 21–47% isolated yields together with a relatively high amount of *trans*-CHD (Table 1). This experiment shows that the limiting factor is not the formation of CHD, as seen in other studies [52]. The molecular catalysts (especially those based on W) were more selective towards AA than the grafted ones. It seems that the transformation of CHD to AA is quicker in the presence of the molecular catalysts, since less CHD intermediate accumulates.

• CHD as the Substrate

When *trans*-CHD was used as the starting substrate, the reaction produced AA in higher isolated yields (59–74%) than when starting from CH within the same time period. 2-Hydroxycyclohexanone, a known intermediate within the postulated oxidation pathway (Scheme 4), [52] was also observed by GC but not quantified. The **C_n_-M** molecular catalysts are highly active (>99% conversion), more than the grafted ones for which the measured conversion is between 71 and 95%. In all the reactions, the W-based catalysts show slightly better performances in terms of activity and AA yields.

• Recycling

Catalyst recovery and recyclability were investigated for the most active grafted catalyst, **MR_12_-W**, and CH as the starting substrate. The IR spectrum of the isolated **MR_12_-W** after the first experiment exhibited no change, except for the presence of additional vibrations attributed to CHD. After the 2nd run, the CH conversion was 54% and the AA yield was 29%. The lower activity after reuse could result from the grafted catalyst inhibition by the presence of CHD within the resin.

• CHK/CHA as the Substrate

**Scheme 5:**
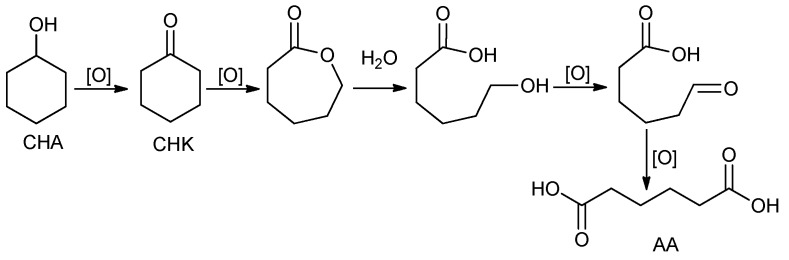
Oxidation steps of CHA and CHK to AA in the presence of H_2_WO_4_ catalyst. Adapted from [2].

In order to mimic a greener industrial process (replacing the traditionally used and environmentally harmful oxidants) the most active molecular catalyst **C_4_-W** (0.025% loading) was also tested for the oxidation of CHK and CHA by H_2_O_2_ (Scheme 5), leading after 24 h to isolated AA yields of 41% and 35% respectively. These results show that **C_4_-W** is less active than H_2_WO_4_ and certain Dawson POMs (58 and 59% with 0.5–2% cat loading with CHA as the substrate) [73], quite similar to (NH_4_)_x_A_y_PMo_12_O_40_ (A^n+^ = Sb^3+^, Bi^3+^, Sn^2+^) species with CHK as the substrate and better with CHA with a lower POM/substrate ratio [74] but more active than other described POMs (see Table 1), and, therefore, also seems promising for the solvent-free oxidation of CHK and CHA [35].

## 3. Materials and Methods

### 3.1. Materials

1-Hexyl-3-methylimidazolium bromide (Aldrich, Saint Louis, MO, USA), H_3_PMo_12_O_40_·26H_2_O (99.9% Merck, Kennallsburg, NJ, USA), H_3_PW_12_O_40_·15H_2_O (purum, Aldrich), 1-methylimidazole (Aldrich), bromobutane (Aldrich), bromododecane (Aldrich), Merrifield resin (Aldrich), EtOH (Aldrich), DMF (Aldrich), dichloromethane (Aldrich), Acetone (Aldrich), Cyclohexene (Aldrich), 35% H_2_O_2_ (Acros, Geel, Belgium) were used as received

### 3.2. Physical Methods

Elemental analyses (EA) were provided by the Analytical Services Laboratory of LCC, Toulouse. The thermogravimetric analyses (TGA) were performed on a SETARAM TGA 92-16.18 thermal analyzer. The sample was placed into an alumina crucible and heated at 0.83 K·s^−1^ in a reconstituted air flow from 25 °C to 600 °C. An empty crucible was used as a reference. The POM percentage in the grafted species was calculated from the percentage of residue measured in the TGA traces divided by the molar mass of PM_12_O_38.5._

Infrared spectra were recorded using the ATR technique with a Perkin Elmer FTIR/FIR 400 spectrometer.

A Bruker Avance DPX-300 spectrometer was used to record ^31^P NMR spectra in the solution at 121.5 MHz. All solid-state ^31^P NMR experiments were recorded on a Bruker Avance 400 spectrometer equipped with a 3.2 mm probe. Samples were spun at 16 kHz at the magic angle using ZrO_2_ rotors. For ^31^P MAS single pulse experiments, small flip angle (~30°) were used with recycling delays of 20 s. ^31^P-CP/MAS spectra were recorded with a recycle delay of 3 s and contact times of 2 ms. ^31^P chemical shifts were referenced to an external 85% H_3_PO_4_ sample.

All the catalytic reactions were followed by gas chromatography on an Agilent 6890 A chromatograph equipped with FID detector, a HP5-MS capillary column (30 m × 0.25 mm × 0.25 μm) and automatic sampling, or on a Fisons GC 8000 chromatograph equipped with FID detector and with a SPB-5 capillary column (30 m × 0.32 mm × 0.25 μm).

The GC parameters were quantified with authentic samples of the reactants and products. The CH conversion, the CHD formation, and the CHO conversion/formation were calculated from calibration curves (r^2^ = 0.999) relative to an internal standard (acetophenone). Acetophenone and CHD are present both in the organic and in the water phase at the end of the catalytic experiments. Therefore, extraction with AcOEt was carried out before the GC analysis. The AA yield was calculated from the isolated mass recovered from the filtered reaction mixture at the end of the reaction.

### 3.3. Preparative Part

• Molecular Catalysts

1-Hexyl-3-methylimidazolium bromide (1.65 mmol, 0.41 g) was mixed with aqueous H_3_PM_12_O_40_ (M = Mo or W) (0.54 mmol, 1.0 g H_3_PMo_12_O_40_ or 1.55 g H_3_PW_12_O_40_) and stirred for 2 h. Yellow (C_6_-Mo) or white (C_6_-W) solids precipitated immediately. The solid was filtered, washed with acetone and ether and dried in air.

The preparation of the C_4_-M (M = Mo or W) catalysts involved the synthesis of the corresponding ionic liquid [75]. 1-methylimidazole (0.05 mol, 4.1 g) and bromobutane (0.05 mol, 6.85 g) were stirred at 70 °C for 24 h in EtOH (20 mL). After the reaction, EtOH was removed by evaporation under reduced pressure. The formation of the corresponding ionic liquid BMImBr was confirmed by IR [76]. BMImBr (4.4 g, 0.02 mol) was directly mixed with aqueous H_3_PM_12_O_40_ (M = Mo or W) (6.67 × 10^‒3^ mol) and stirred for 2 h. A yellow (C_4_-Mo) or white solid (C_4_-W) precipitated immediately. The solid was filtered, washed with acetone and ether and dried in air.

**C_4_-Mo**: Yield: 10.3 g (69%). EA: calculated for C_24_H_45_Mo_12_N_6_O_40_P (%), C: 12.9, H: 2.0, N: 3.6, found (%), C: 12.9, H: 1.9, N: 3.7. IR-ATR (cm^−1^): 1060, 945, 867, 763, 732. ^31^P NMR (ppm): −4.15.

**C_4_-W**: Yield: 18.6 g (85%). EA: calculated for C_24_H_45_W_12_N_6_O_40_P (%), C: 8.7, H: 1.4, N: 2.6, found (%), C: 8.9, H: 1.5, N: 2.7. IR-ATR (cm^−1^): 1053, 945, 870, 775, 722. ^31^P NMR (ppm): −15.5.

**C_6_-Mo**: Yield: 11.4 g (74%). EA: calculated for C_30_H_57_Mo_12_N_6_O_40_P (%), C: 15.5, H: 2.4, N: 3.6, found (%), C: 16.0, H: 2.3, N: 3.8. IR-ATR (cm^−1^): 1078, 970, 885, 771, 732. ^31^P NMR (ppm): −4.15.

**C_6_-W**: Yield: 17.6 g (78%). EA: calculated for C_30_H_57_Mo_12_N_6_O_40_P (%), C: 10.7, H: 1.7, N: 2.5, found (%), C: 10.8, H: 1.6, N: 2.5. IR-ATR (cm^−1^): 1072, 969, 885, 777, 726. ^31^P NMR (ppm): −15.5.

• Grafted Catalysts

The Merrifield resin (1.4–1.6 mmol Cl/g, 5.6–6.4 mmol, 4 g), 2-methylimidazole (0.05 mol, 4 g), and Na_2_CO_3_ (0.01 mol, 1.3 g) were stirred in DMF (20 mL) at 80 °C during 24 h [77]. After washing the resin with dichloromethane (to remove the organic compounds) and then in water until neutral pH, the resin was dried in air. This resin was then placed with 5 mL bromobutane (**MR_4_-**) or bromododecane (**MR_12_-**) and stirred for 72 h at 80 °C. The resin was then washed with dichloromethane, ethanol, and acetone. Finally, it was treated with 5 mL of aqueous solution of H_3_PMo_12_O_40_ (5 × 10^−5^ mol, 0.09 g) or H_3_PW_12_O_40_ (5 × 10^−5^ mol, 0.15 g). After filtration and washing with distilled water and acetone, around 4 g of light yellow resins (**MR_4_-Mo**, **MR_4_-W**, **MR_12_-Mo**, **MR_12_-W**) were obtained.

**MR_4_-Mo:** EA: found (%), C: 59.5, H: 6.8, N: 5.0. TGA (residue) 11.9%. Mo loading (μmol POM/g of polymer): 66.7. IR-ATR (cm^−1^): 1060, 953, 878, and 802. ^31^P NMR (ppm): −3.5.

**MR_4_-W** EA: found (%), C: 55.9, H: 6.0, N: 4.5. TGA (residue) 3.5%. W loading (μmol POM/g of polymer): 12.3. IR-ATR (cm^−1^): 1079, 975, 894, and 811. ^31^P NMR (ppm): −15.1.

**MR_12_-Mo** EA: found (%), C: 40.9, H: 4.1, N: 4.3. TGA (residue) 9.99%. Mo loading (μmol POM/g of polymer): 55.6. IR-ATR (cm^−1^): 1060, 952, 876, and 794. ^31^P NMR (ppm): −1.9, −3.5.

**MR_12_-W** EA: found (%), C: 30.0, H: 3.0, N: 3.5. TGA (residue) 5.3%. W loading (μmol POM/g of polymer): 18.9. IR-ATR (cm^−1^): 1078, 974, 893, and 797. ^31^P NMR (ppm): −12.4, −15.2.

### 3.4. Swelling Test for MR

The MR (0.5 g) was placed in a glass test tube with stopper and CH or CHO was added. The reaction mixture was heated at 60 °C for 6 h. The mixture was filtered and the recovered resin was dried in air and weighted.

### 3.5. General Procedure for Catalytic Oxidation to AA

#### 3.5.1. CH Oxidation (Procedure A)

A round-bottom flask equipped with a magnetic stirring bar and a reflux condenser was charged with CH (80 mmol, 8 mL), acetophenone (3.5 mmol, 0.4 mL), and catalyst (0.02 mmol of molecular catalyst or 0.06 g of grafted catalyst). After the stirred reaction mixture reached 60 °C, H_2_O_2_ (320 mmol, 31.1 g, 35%) was added and stirring was continued for 72 h. The final mixture was filtered and the filtrate was left to stand in the refrigerator for one day. The resulting white precipitate was filtered and identified as AA by IR, NMR, and DSC.

#### 3.5.2. CHO Oxidation (Procedure B)

Similar to *procedure A*, with a reaction time of 24 h and 2 eq of H_2_O_2_ (160 mmol, 35%).

#### 3.5.3. CHD Oxidation (Procedure C)

Similar to *procedure A*, with a reaction time of 72 h and 2 eq of H_2_O_2_ (160 mmol, 35%).

#### 3.5.4. Recycling of the Resins

The resins were isolated at the end of the catalytic reaction, washed with diethylether at room temperature, and dried under ambient air.

#### 3.5.5. CHK or CHA Oxidation (Procedure D)

A round-bottom flask equipped with a magnetic stirring bar and a reflux condenser was charged with cyclohexanone (80 mmol, 7.85 g) or cyclohexanol (80 mmol, 8.0 g) and 10 mL of water. Acetophenone (3.5 mmol, 0.4 mL) was added as an internal standard and the catalyst (0.02 mmol) was added. After the stirred reaction mixture reached 80 °C, H_2_O_2_ (320 mmol, 31.1 g, 35%) was added and stirring was continued for 24 h. The homogeneous solution was allowed to stand at 0 °C for 12 h, and the resulting precipitate was separated by filtration and washed with 20 mL of cold water. The product was dried in a vacuum to give AA as a solid.

## 4. Conclusions

Immobilization of the Keggin phosphomolybdate and phosphotungstate on functionalized Merrifield resins has led to the development of new heterogeneous catalysts for AA production in a one-step procedure from CH. Different intermediates were detected and the key steps could be determined by running comparative oxidation tests form each one of the possible intermediates. The substrate itself is able to swell the MR allowing access to all catalytic sites without the use of the classical swelling organic solvents. This atom economical methodology obeys a few of the green chemistry principles (*greener oxidant*, no phase transfer catalysts, *no organic solvents added* (safer reagents and atom economy), *minimal catalyst loading* and recyclability). The developed protocol is straightforward, safe, and environmentally benign and can be extended to a variety of other POM-based catalyzed oxidations.

## Figures and Tables

**Table 1:** Reaction conditions for the preparation of adipic acid from CH, CHO, CHD, CHK, CHA (see footer for abbreviations)—Some literature data with Mo, W-based polyoxometalates (POMs) and mesoporous catalysts.

Sub.	Cat.	Cat/Sub Loading	Ox. Agent (eq)	T (°C)	Time (h)	AA Yield (%)	Sub Conv (%)	Ref.
CH	Na_2_WO_4_·2H_2_O [CH_3_(*n*-C_8_H_17_)_3_N]HSO_4_	1%	A	75–90	8	90	>99	[40]
CH	Na_2_WO_4_ + ILs	2.5%	4.4 A	reflux	10	100	89–97	[55]
CHK CHA	H_2_WO_4_ + ILs	1%	3.3 A 4.4 A	90	20	90	>99	[35]
CH	H_2_WO_4_ + IL	2%	4.4 A	73–87	12	85–96	n.d.	[56]
CH	H_4_SiW_12_O_40_	2%	5 A	US (25 kHz)	4	92		[57]
CHA	(C_4_H_9_N)_3_[PMo_12_O_40_]	0.02%	3 A	reflux	17	18	>99	[39]
CHA CHK	H_3_PMo_12_O_40_ NiPMo_12_O_40_ CoPMo_12_O_40_	0.5%	1 A	90	14	17–30 17–32	>99	[36]
CHK CHA	H_3−2x_Ni_x_PMo_12_O_40_ (NH_4_)_3−2x_Ni_x_PMo_12_O_40_ (x = 0.0–1.5)	0.1–0.4%	1 A	90	20	34–45	>99	[38]
CHK CHA	H_3−2x_Co_x_PMo_12_O_40_	0.15–0.66%	1 A	90	20	30–50	>99	[37]
CHD CHO CHK CHA	Ti-Y zeolites	sub/cat mass ratio = 9.7	4 A	80	24	13–39 44 78 70	24–60 59 36	[42]
CH	Ti-MMM-2 Ce-SBA-15	5 mmol CH 50 mg cat	3.6 A	80	72	10–30	>99	[51]
CH CHO CHD	TAPO-5	62 mmol 0.5 g cat	3.5 A	80	72 24 24	30 40 42	>99 >99 >72	[52]
CHD CHO	Ti-AlSBA-15 + 10 mL CH_3_CN	7 mmol sub 0.1 g	3 B	80	48	50–88 94	56–98 84	[53]
CH		7.8%	3 A	70	24	80	7	[58]
			1.5 B	80	2	10	15	
CHO	Ti(16)Al(DS)SBA + 10 mL CH_3_CN	9.7%	3 B	80	48	94	84	[54]
CHD					24–48	76–96	33–88	
CH		9.7–19.4%	4B				40–84	

CH = cyclohexene, CHA = cyclohexanol, CHK = cyclohexanone, CHO = cyclohexene oxide, A = aqueous H_2_O_2_, B = TBHP in decane AA: adipic acid, US = ultrasound, IL= ionic liquid.

**Table 2:** CHN elemental analyses and metal loading.

EA Data	C (%)	H (%)	N (%)	Cl (%)	M (Mo or W) Loading (µmol POM/g of Polymer) *
**MR**	79.1	6.4	-	14.5	-
**MR_im_**	74.6	8.4	6.9	10.1	-
**MR_4_-Mo**	59.5	6.8	5.0	n.d.	66.7
**MR_4_-W**	55.9	6.0	4.5	n.d.	12.3
**MR_12_-Mo**	40.9	4.1	4.3	n.d.	55.6
**MR_12_-W**	30.0	3.0	3.5	n.d.	18.9

n.d. = not determined, * based on the TG analyses.

**Table 3:** Data of oxidation reactions for AA production starting from different substrates (CH, CHO, CHD) and different catalysts.

	Molecular Catalysts				Grafted Catalysts			
	C_4_		C_6_		MR_4_		MR_12_	
	Mo	W	Mo	W	Mo	W	Mo	W
%Cat *^a^*	0.025				0.004	0.007	0.003	0.001
CH as substrate								
**CH conv.** *^b^*	65	75	61	68	58	61	56	73
AA yield *^c^*	46	61	42	50	33	43	30	51
CHD form *^d^*	15	12	11	17	24	17	19	20
CHO as substrate								
**CHO conv.** *^e^*	>99				>99			
AA yield *^c^*	36	47	32	43	28	33	21	36
CHD form *^d^*	25	26	29	24	28	23	32	35
CHD as substrate								
**CHD conv.** *^f^*	>99				71	94	79	95
AA yield *^c^*	74	72	58	63	46	60	41	56

*^a^* Molar ratio (in percentage) relative to the substrate. *^b^* CH conversion (in %) after t = 3 days, T = 333 K, 4 eq of H_2_O_2_ were added in small portions. *^c^* isolated AA yield (in %). *^d^* CHD formation (%) based on GC. *^e^* CHO conversion (in %) after t = 1 day, T = 333 K, 2 eq of H_2_O_2_ were added at the beginning of the reaction. *^f^* CHD conversion (in %) after t = 3 days, T = 333 K, 2 eq of H_2_O_2_ were added at the beginning of the reaction.
